# Acid-Fast Bacilli in Faeces of Patients Suffering from Joint Disease

**Published:** 1915-12

**Authors:** 


**Acid-Fast Bacilli in Faeces of Patients Suffering from Joint Disease.** Henry Keller and A. J. Moravek, N. Y., Med. Rec. Nov. 20. By animal experiments, the authors corroborate the contention of Rosenburger in 1907 that acid-fast bacilli in the faeces are tubercle bacilli and not smegma bacilli or other comparatively harmless germs. The smegma bacillus is conspicuous by its absence, in the faeces. Shortly before death in guinea pigs and during states of depression in patients, tubercle bacilli tend to disappear from the faeces. In 42 cases of active tuberculosis of the joints, they found tubercle bacilli in the faeces in 30 and in the urine in 2. Of 6 cured cases, none showed bacilli in either excretion. In 18 control cases, none showed bacilli in either. Of 9 originally doubtful cases, 1 showed tubercle bacilli in the faeces but not in the urine. This case proved to be active tuberculous arthritis of the right hip. Of the 8 negative cases, 1 proved to be an ordinary sprain of the knee, 1 a gonorrhoeal arthritis, 3 syphilitic, 2 remain doubtful and 1 has been lost sight of.

Note. Unless further contradictory evidence is adduced, this corroboration of the views of Rosenburger and of M. Solis Cohen and others, deserves wide attention. It justifies the dictum that there is no such thing as a closed tuberculosis unless the process has also become strictly latent and encapsulated. It points out a neglected route of infection and demands the most careful disinfection of the stools—not to mention the urine—of all active tuberculous cases. It adds a detail of great importance in rendering the consumptive or otherwise tuberculous person, cleanly.

